# Cellular Liver Regeneration after Extended Hepatic Resection in Pigs

**DOI:** 10.1155/2009/306740

**Published:** 2009-03-31

**Authors:** Ruth Ladurner, Frank Traub, Martin Schenk, Alfred Königsrainer, Jörg Glatzle

**Affiliations:** Department of General, Visceral and Transplant Surgery, University Hospital Tübingen, Hoppe-Seyler-Street 3, D-72076 Tübingen, Germany

## Abstract

*Background*. The liver has an enormous capacity to regenerate itself. The aim of this study was to evaluate whether the regeneration is due to hypertrophy or hyperplasia of the remnant liver after extended resection and whether a portosystemic shunt is beneficial. *Material and methods*. An extended left hemihepatectomy was performed in 25 pigs, and in 14 after performing a portosystemic shunt. During follow up, liver regeneration was estimated by macroscopic markers such as liver volume and size of the portal fields [mm^2^] as well as the amount of hepatocytes per portal field and the amount of hepatocytes per mm^2^. *Results*. Regardless of the operation procedure, the volume of the remnant liver increased about 2.5 fold at the end of the first week after resection. The size of the portal fields increased significantly as well as the number of hepatocytes in the portal fields. Interestingly, the number of hepatocytes per mm^2^ remained the same. *Conclusion*. After extended resection, liver regeneration was achieved by an extensive and significant hyperplasia of hepatocytes within the preexisting portal fields and not by de novo synthesis of new portal fields. However, there was no difference in liver regeneration regarding the operation procedure performed with or without portosystemic shunt.

## 1. Introduction

The liver has a remarkable regenerative capability and is able to restore to its original weight and size after resection of approximately 80%. This regenerative capacity is a determining factor in modern liver surgery, such as in the case of portal vein embolisation of the tumor-bearing segment in order to augment the remnant liver if small in size [1]. The major concern here is the size of the remaining liver mass. A small-sized remnant liver is likely to be functionally inadequate and susceptible to poor regeneration, hepatic insufficiency, and finally liver failure. Therefore, improved knowledge on how to estimate the capacity of liver regeneration is of critical importance for extended liver surgery.

Knowledge on the proliferative capacity of hepatocytes and the mechanisms involved in liver regeneration has evolved very rapidly during the last few years. In great part, but not exclusively, these advances are based on the development of genetically-engineered mice in which specific genes are inserted or deactivated. However, the mechanism determining liver regeneration is not as yet completely understood. 

Scant data are presented in literature on large animal models from the functional and morphological site of liver regeneration. It is so far unknown whether the underlying cellular mechanism of liver regeneration is due to hypertrophy or hyperplasia of the portal fields. 

The present study was therefore designed for a pig model in order to explore whether liver regeneration is due to hypertrophy or to hyperplasia of the remnant liver tissue after extended hepatic resection and whether regeneration is more effective with portosystemic shunt, which is supposed to attenuate portal hyperperfusion after liver surgery.

We investigated liver regeneration in close weekly intervals by correlating the size of the portal fields, the number of hepatocytes per portal field, and the number of hepatocytes per mm^2^ by time using light microscopy and computed analyzing software.

## 2. Materials and Methods

### 2.1. Animals

An extended left hemihepatectomy (approximately 75% of liver volume), was performed in healthy female, German landrace pigs, 6–8 weeks old and weighing 25–35 kg. For blood and vessel procurement (infrarenal aorta), performed to create a portosystemic H shunt in pigs undergoing liver resection, male sibling pigs were used as donors. The project and study design were approved by the Austrian Federal Animal Investigation Committee, and animals were treated in accordance with the National Institutes of Health guidelines.

### 2.2. Experimental Procedure

Animals were divided into two groups. The control group (*n* = 11 pigs) underwent only an extended left hemihepatectomy (approximately 75% of liver volume). The shunt group (*n* = 14 pigs) received a side-to-side portosystemic H-shunt as an interposition graft (7 × 8 mm aortic allograft, diameter 7-8 mm, 6 running suture after partial clamping of the portal and infrahepatic caval veins) between the portal vein and infrahepatic vena cava before extended liver resection. 

After surgery, the animals were weaned from anaesthesia and extubated. Peripheral venous catheters were left in place for additional fluid resuscitation during the first days after surgery. Pigs resumed oral feeding ad libitum after recovery. Low-dose heparin (2 mg/kg/d) was administered in the shunt group. The animals were observed regularly under sedation with ketamine (5–10 mg/kg intramuscularly). Doppler ultrasound was performed to assess the patency of the shunt and flow rates (mL/min) in the portal vein and in the right lateral artery. For data analysis, all *n* ** = **14 pigs in the shunt group and *n* ** = **11 pigs in the control group were used. Specimens of the liver were taken weekly up to the third week in both groups. The male sibling pigs also underwent an extended left hemihepatectomy in order to measure the liver volume being resected, the remaining liver tissue after extended left hemihepatectomy as well as the total liver volume at the time point of the operation. 

The liver volume being resected in the pigs received an extended hemihepatectomy either with or without H-shunt and was measured at the time of the operation and compared with the liver volume being resected in the male sibling pigs that served as blood vessel donors. The liver volume of the remnant regeneration liver was measured at the end of the experiment. Since the total liver volume and the remnant liver volume at the time of surgery could not be measured in pigs with extended liver resection, data from the male sibling pigs were used for comparison. The liver volume was measured by water displacement and is given in millilitres (mL). 

### 2.3. Light Microscopy and Evaluation of Regeneration

For histomorphological evaluation, all liver specimens were fixed in 4% phosphate buffered paraformaldehyde. Liver specimens were subsequently dehydrated and embedded in paraffin wax to process sections at a thickness of 6 *μ*m. The sections were conventionally stained by hematoxylin and examined using computer-assisted brightfield miscroscopy, with 50× magnification. To evaluate the histological regeneration of the remnant liver, the area of the periportal fields, the number of hepatocytes within the periportal fields, and the number of hepatocytes per mm^2^ were analyzed using Leica Quantimet 500 software. In summary, the periportal fields were surrounded, and the area of the periportal field was measured in mm^2^. Thereafter a colorthreshold was set in order to demarcate the nucleus of the hepatoctes. The number of hepatocytes within the marked periportal field was calculated by the software system and controlled for accuracy by the investigator. In order to measure the density of the hepatocytes within the periportal field, the quotient of hepatocytes per area of the periportal field was calculated and expressed as hepatocytes per mm^2^.

## 3. Results

### 3.1. Survival Rates of Animals after Extended Liver Resection

In general, survival rates did not differ between the two groups. Three animals in the control group and two in the shunt group developed ascites at the end of the first week after surgery. Nevertheless, none of the animals in either group showed splenomegaly, congestion of the bowel, or thrombosis of the portal system. 

### 3.2. Blood Flow in the Portal Vein and Hepatic Artery after Liver Regeneration

The blood flow in the hepatic artery increased slightly, but not significant in animals after extended liver resection without shunt: 49 ± 8 mL/min before resection and 79 ± 13 mL/min after resection. In the group with H-shunt, the increase was also not significant: 50 ± 8 mL/min before resection and 55 ± 8 mL/min postoperatively. 

However, in animals with H-shunt the portal blood flow decreased significantly: 1203 ± 187 mL/min before resection and 370 ± 32 mL/min after resection (*P* < .005). In the animals without shunt, the portal blood flow was not altered (717 ± 155 mL/min before resection and 534 ± 61 mL/min after resection, not significantly different).

### 3.3. Macroscopic Liver Regeneration

After resection of 75% of the liver volume, the regeneration rate of the right lateral segment and segment I—defined as remnant liver volume at follow-up/remnant liver volume after surgery—was not significantly different between the two groups (Table 1).

### 3.4. Histopathological Analysis of Liver Regeneration

#### 3.4.1. Size of Portal Fields (Figure 1)

The size of the portal fields increased significantly after extended liver regeneration in both groups undergoing surgery with or without portosystemic shunt. The size of the portal fields increased about 3-fold within 3 weeks regardless of the operation procedure. The correlation analysis revealed a highly significant correlation with a coefficient of *r* ^2^ ** of 0.992 and 0.974 for animals resected with and without portosystemic shunt, respectively, (portal; field [mm^2^]; animals with shunt before resection: 1 ± 0.4; 1 week after resection: 1.8 ± 0.9*; 3 weeks: 2.9 ± 1.3*, *P < .05; animals without shunt before resection: 1.1 ± 0.3; 1 week after resection 1.8 ± 0.7*; 3 weeks: 2.9 ± 1.7*, *P < .05).

#### 3.4.2. Hepatocytes in Portal Fields (Figure 2)

The number of hepatocytes within the portal fields increased significantly over time, at approximately 2.6 fold within 3 weeks, regardless of the operation procedure with or without portosystemic shunt. The correlation coefficient was in both groups high, measuring *r* ^2^ = 0.935 and *r* ^2^ = 0.909 in pigs resected with or without shunt, respectively, (hepatocytes/portal field, animals with shunt before resection: 3438 ± 1281; 1 week after resection: 5831 ± 3419*; 3 weeks: 8793 ± 3690*, *P < .05; animals without shunt before resection: 3053 ± 1096; 1 week after resection: 4580 ± 2007*; 3 weeks: 8014 ± 4809*, *P < .05).

#### 3.4.3. Hepatocytes per Area (Figure 3)

Interestingly, the number of hepatozytes per mm^2^ remained very consistent over the time after liver resection and was not different between the groups resected with or without portosystemic shunt (hepatocytes per mm^2^, animals with shunt before resection: 3195 ± 36; 1 week after resection: 3145 ± 38; 3 weeks: 2999 ± 46; animals without shunt before resection: 2863 ± 88; 1 week after resection: 2625 ± 118; 3 weeks: 2676 ± 83, not significant). 

## 4. Discussion

The ability of the adult liver to restore its function and mass after injury or extended resection is unique. Nevertheless, hepatic failure can closely be associated with impaired liver regeneration, caused by the damage of hepatic sinusoids resulting from excessive blood flow and transient portal hyperperfusion [2–4]. The critically sized graft with a calculated liver weight of less than 20% might be functionally adequate if we could protect it from portal flow-related injuries by maintaining intrasinusoidal hemodynamics within a “physiological” range that would not harm Kupffer cells or the endothelial lining cells [5]. Partial diversion of portal flow to the systemic circulation through a portocaval shunt might therefore be a reasonable approach in reducing the risk of these injuries occurring. A portocaval interposition graft or H-shunt between portal vein and infrahepatic vena cava in our animal model was performed to reduce portal flow through the reduced vascular network of the liver remnant after resection and to avoid portal hypertension and irreversible injuries. The interposition graft permits a reduced hepatopedal flow and allows the passage of necessary regeneration factors from the intestinal tract, promoting hepatic proliferation in the remnant liver. Finally, when liver regeneration is completed, the shunt can be easily occluded by interventional radiological embolization. Nevertheless, extended hepatic resection with or without shunt in our animal study did not reveal significant differences between the two groups, indicating comparable liver regeneration. Additionally, Man et al. [2] suggest that transient portal hypertension and excessive hepatic blood flow are found in the first 30 minutes after reperfusion of the small-for-size liver graft in a rat model, and, although the portal hemodynamic changes are transient, the small-for-size graft injury is continuous and progressive, suggesting that irreversible sinusoidal damages occurred in the early phase after reperfusion.

Whether regeneration represents simple hyperplasia of various liver elements or requires hypertrophy and recapitulation of the developing genetic liver program is as yet unknown [6]. In addition, a dramatic change in the portal blood flow is an organ-specific characteristic inducing a rapid modification in liver volume. Microscopic analysis of the liver at different time intervals after extended hepatic resection represents the most complete data set to date for examining hyperplasia/hypertrophy during regeneration of portal fields.

Very little is known or has been published on histomorphological liver regeneration of a mammalian liver similar in morphology and size to the human liver. We studied a large animal model comparable to humans [7–9] that offers information more relevant to human biology than the commonly employed mouse and rat models. In order to investigate the association between liver regeneration and changes in the size of portal fields, hepatocytes per portal field as well as density of the hepatocytes, we applied histological analysis supported by a powerful computational analysis at different time intervals and up to 3 weeks after extended liver resection. Additionally, liver regeneration was analyzed histomorphologically in animals resected with and without portosystemic shunt. The pig model of extended liver resection with and without portosystemic shunt was already discussed and published by our group in 2006 [10]. This procedure was performed to induce compensatory hyperplasia/hypertrophy, characterized by proliferation of normal quiescent hepatocytes [11, 12]. Based on observations made concerning small-sized liver transplant recipients and patients with massive liver resection, hepatic decompression was performed in a separate experimental group by H-shunt between portal- and caval vein as an additional procedure to enhance liver regeneration [13, 14]. This approach was based on the hypothesis that abrogation of increased portal pressure and shear stress following liver resection would prevent continuous liver damage and promote regeneration. 

The liver receives its dual blood supply from the portal vein (approximately 80%) and the hepatic artery (approximately 20%), and blood flow from the two vessels follows a different pattern intrahepatically, though both vessels share the common draining vessel of a terminal hepatic venule in the centre of the portal field [15–20]. A decrease in portal venous flow is compensated for by an increase in hepatic arterial flow, the so-called hepatic artery buffer response, first described by Lautt et al. in the 1980s [21, 22]. We observed a significant decrease of portal blood flow in the shunt group but not in the group without shunt, and in both groups the increase in the hepatic artery flow was not significant. 

Nevertheless, we did not observe clinically evident liver damage nor a significant influence on liver regeneration between the two groups and thus concluded that a shunt after 75% hepatectomy does not influence portal hypertension and regeneration in pigs [10]. Wege et al. (2007) described mitotic activity and telomerase activation, correlated with mitotic hepatocytes, which was significantly increased in pigs after 70%–80% liver resection or liver resection and TIPS alone. However, no significant differences were observed between the two resection groups [9]. It is known that quiescent hepatocytes become proliferative and restore liver functional capacity as well as liver mass in cases of tissue resection. Portosystemic shunt in a small-sized liver remnant is supposed to diminish the damage caused by portal hyperperfusion of a small liver remnant. However, we found that, after 70%–80% of liver resection in pigs, the amount of metabolic work needed to meet the functional requirements of the animals was reached. Damage by portal hyperperfusion was not pronounced and not significant.

Other authors have described [23] that hepatocytes divide once or twice and then return to quiescence during liver regeneration after 70% hepatectomy. Although we did not focus on the replication of hepatocytes, we did observe that the increase of hepatocytes per portal field after resection did slow slightly. The increase, however, was always significant immediately following resection to three weeks later. Nevertheless, the amount of hepatocytes per area (mm^2^) showed no change in both groups. Similar findings have been previously observed by intravital microscopy of morphological features in regenerating rat livers [20]. 

Despite different hemodynamics, the histological process of hepatic regeneration (following 75% of liver resection) in both study groups was very similar. Nevertheless, due to a sudden reduction in hepatic volume, hepatic hemodynamic changes in both groups were dramatic, which may have contributed to a rapid activation of liver proliferation. Another factor that probably affected hepatic regeneration in our model was aging. Our animals were 6-to-8-week old pigs. There is some evidence to indicate that the proliferative response in livers of older animals seems to be attenuated via a mechanism mediated, for example, by c-myc promoter blocking [24].

In conclusion, after extended liver resection, restoration of liver volume can be accomplished by an extensive and significant hyperplasia of hepatocytes within the pre-existing portal fields, inducing significant hypertrophy of the portal fields. A denovo synthesis of new portal fields seems unlikely, since an increase in small portal fields was not observed after liver resection. However, the hypertrophy of the portal fields means a longer distance for oxygen diffusion within the portal fields. No difference in histomorphological liver regeneration was observed after extended liver resection in animals that underwent surgery with or without portosystemic shunt. 

## Figures and Tables

**Figure 1:**
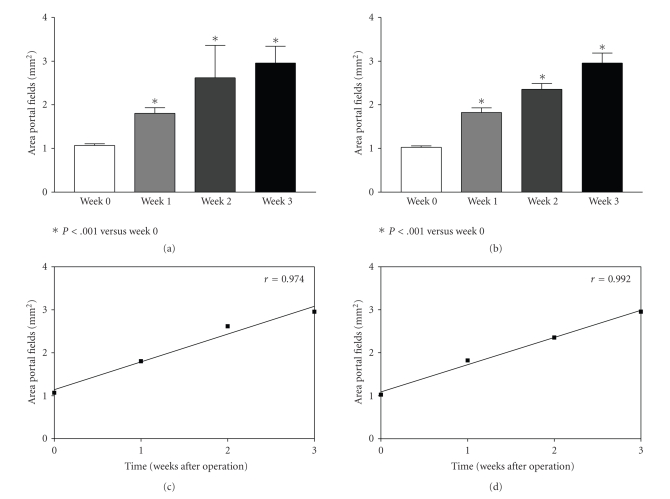
The areal increase of the pre-existing portal fields is expressed in Figure 1 in animals operated without (Figures 1(a) and 1(c)) and with portosystemic H-shunt (Figures 1(b) and 1(d)). The areal increase of the portal fields is shown in relation to the postoperative time (weeks) and is expressed as mean ± SEM in Figures 1(a) and 1(b) and as correlation coefficient in Figures 1(c) and 1(d). The areal increase of the portal fields was highly significant after the first week (*P* < .001).

**Figure 2:**
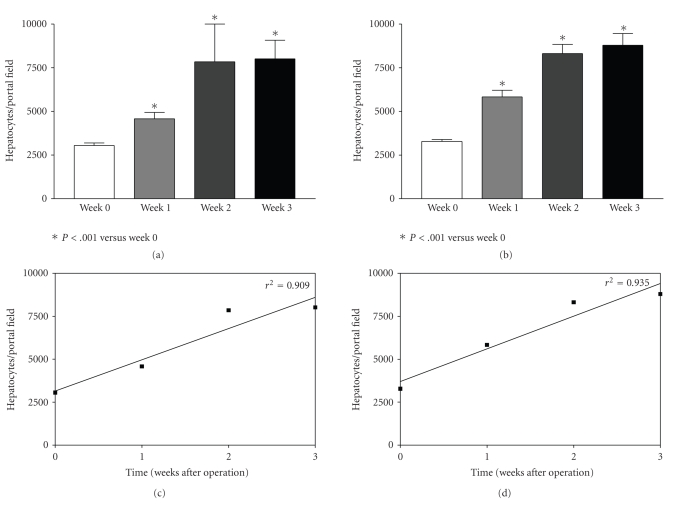
The increase of hepatocytes in the pre-existing portal fields is expressed in Figure 2 in animals operated without (Figures 2(a) and 2(c)) and with portosystemic H-shunt (Figures 2(b) and 2(d)). The increase of hepatocytes is shown in relation to the postoperative time (weeks) and is expressed as mean ± SEM in Figures 2(a) and 2(b) and as correlation coefficient in Figures 2(c) and 2(d). The increase of hepatocytes in the portal fields was highly significant after the first week (*P* < .001).

**Figure 3:**
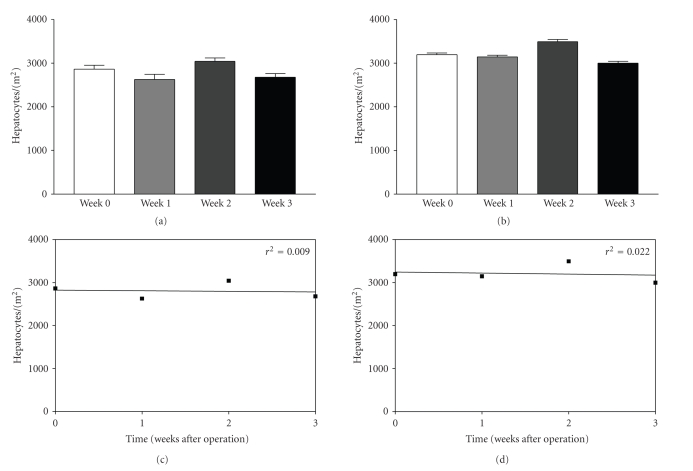
The density of hepatocytes per mm^2^ is expressed in Figure 3 in animals operated without (Figures 3(a) and 3(c)) and with portosystemic H-shunt (Figures 3(b) and 3(d)). The density of hepatocytes is shown in relation to the postoperative time (weeks) and is expressed as mean ± SEM in Figures 3(a) and 3(b) and as correlation coefficient in Figures 3(c) and 3(d).

**Table 1:** Regeneration of liver volume (liver volume at follow up/remnant liver volume after surgery).

	With shunt	Without shunt
1st postoperative week	2.2 ± 0.1	3.0 ± 0.7
3rd postoperative week	6.5 ± 0.5	5.8 ± 0.8
